# COVID-19: A time for renewed recognition of science

**DOI:** 10.1016/j.ebiom.2020.102869

**Published:** 2020-06-21

The world's largest annual celebration of biomedical science takes place on June 11, 2020. Founded by the UK Institute of Biomedical Science, Biomedical Science Day aims to celebrate the countless ways biomedical research enriches our health care. As the COVID-19 pandemic continues to immobilise everyday life, this special day is a good chance to reflect on the tremendous efforts biomedical scientists have exerted in tackling this crisis.

“Following the science” has been the mantra repeated by governments across the world during this pandemic, and scientists have had an instrumental role by continually informing policy makers. However, this mantra is not without its challenges. The scientific method typically involves careful observation, rigorous scepticism, and an iterative self-correction process that is not necessarily conducive to formulating policy in a rapidly changing global health crisis. On May 20, 2020, Sir Venkatraman Ramakrishnan, the president of the Royal Society, stated that “scientists should stick to advice and then it is for government to accept the advice and decide what to do with it.” This statement was in response to growing unease that governments might start attributing blame to scientists for providing incorrect advice during this crisis. After all, hindsight is an exact science. As lockdowns are gradually eased, maintaining an effective working relationship between government and scientists will be crucial for tracking and tracing new cases and devising therapeutic strategies to minimise the chances of a second wave.

As this editorial goes to press, the worldwide number of severe acute respiratory syndrome coronavirus 2 (SARS-CoV-2)-infected individuals now exceeds 5 million, precipitating a swift and collaborative response from the scientific community. The rapid publication and dissemination of robust, peer-reviewed research has been crucial to inform these efforts. On January 30, 2020, less than one month after patients first presented with viral pneumonia in Wuhan, China, The *Lancet* published the first genomic characterisation of SARS-CoV-2. This publication initiated a scramble to understand the virus and the complex pathophysiology of COVID-19. Indeed, over 18 000 publications relating to COVID-19 have been indexed in PubMed since January, 2020; a number that does not include the deluge of studies deposited on non-peer reviewed preprint platforms. Sensitive tests for active infection have been quickly developed and implemented, followed by antibody tests to assess if an individual has had exposure to the virus. Understanding why some individuals fare worse is also an active area of research with scientists trying to look for genetic and environmental clues that might make an individual more susceptible to this novel coronavirus.

This critical research is expensive, and emergency funding initiatives have been established across the world. On May 4, 2020, the EU held an online pledging conference in which 40 countries and donors took part. More than US $8 billion was promised to help develop a SARS-CoV-2 virus vaccine and fund research for the diagnosis and treatment of the disease. In the USA, the National Institutes of Health has launched several initiatives, including the Rapid Acceleration of Diagnostics (RADx) initiative and the Accelerating COVID-19 Therapeutic Innovations and Vaccines (ACTIV) public-private partnership. RADx has $1.5 billion federal stimulus funding and ACTIV aims to develop a collaborative framework to fast-track vaccine and drug candidates and streamline clinical trials. Vaccine development is already showing promise and, on May 22, 2020, The *Lancet* published the first-in-human phase 1 clinical trial for a COVID-19 vaccine from China. The study reported that a recombinant adenovirus type-5 vectored COVID-19 vaccine expressing the spike glycoprotein of a SARS-CoV-2 strain was tolerable and immunogenic at 28 days post-vaccination in healthy individuals. Moderna (MA, USA) also recently announced positive interim phase 1 data for its mRNA vaccine (mRNA-1273) against SARS-CoV-2. The rapidity of vaccine development has been astonishing, but only further testing will confirm if these promising findings will translate into successful vaccines that can be rolled-out around the world.

For scientists whose research is not directly linked to the response to COVID-19, this pandemic has imposed a range of different challenges. Many research laboratories have either been repurposed to focus on COVID-19 or shut for all but the most essential experiments meaning research has stalled. This situation is not ideal, especially when research output is a key determinant for extending short-term contracts. According to the UK's Office for National Statistics, around three-quarters of education and scientific enterprises have taken measures to reduce hours, lay off, or furlough staff to cope with the financial pressures of the lockdown. For those working overseas, lockdown has trapped them away from home and separated them from their families. These factors are likely to have a profound effect on physical and mental wellbeing. On June 1, 2020, The *Lancet Psychiatry* published a position paper setting out immediate priorities and longer-term strategies for mental health science research to address the psychological consequences of this pandemic. It is hoped that efforts such as these will help to support the mental wellbeing of individuals that have been affected.

As laboratories are gradually being given permission to reopen, scientists wait with trepidation as plans are devised to do this as safely as possible. The term “new normal” is being used to describe life after lockdown, but how might this pandemic shape future research and what might post-lockdown life look like for scientists? Practically, reintroducing scientists back to the lab will likely involve strict distancing measures. Wearing face masks, limiting the number of people in communal areas, and shifting (or staggering) working days to avoid a typical rush hour might be enforced. Scientifically, funding bodies might re-direct money towards infectious disease research to better prepare for future pandemics. Virtual conferences might become more prevalent in response to the demise of airlines and reluctance to travel internationally. This change might prove to be a more inclusive system as they can be virtually attended by more people because of reduced fees and the absence of logistical constraints. Societally, this crisis has highlighted the importance of scientists and the need to share knowledge and data. Perhaps this change will lead to a renewed recognition of science in society and increased funding to nurture this partnership.

Whatever the future holds, *EBioMedicine* would like to take this opportunity to thank the brilliant scientists throughout the world whose efforts are making a difference. From processing tests and performing research, to ensuring rapid peer review and creating an important dialogue with the public to ensure opinions have a factual basis, scientists have been vital. Biomedical Science Day is an opportunity to celebrate these achievements and to applaud the work that you do!

*EBioMedicine*

